# Differentially Methylated Regions in Human Rhombic Lip Compartments Are Enriched in Putative Active Enhancers, Human Accelerated Regions, and Medulloblastoma Copy Number Aberrations

**DOI:** 10.1007/s12311-026-02057-4

**Published:** 2026-07-21

**Authors:** Xinghan Sun, Soumya Menon, Paul Wambo, Ilinca Lungu, Birth Defects Research Laboratory, Kimberly A. Aldinger, Shraddha Pai

**Affiliations:** 1 Ontario Institute for Cancer Research, Toronto, Canada; 2 Department of Medical Biophysics, University of Toronto, Toronto, Canada; 3 University of Waterloo, Waterloo, Canada; 4 Department of Pediatrics, University of Washington, Seattle, USA; 5 Norcliffe Foundation Center for Integrative Brain Research, Seattle Children’s Hospital, Seattle, USA; 6 Department of Neurology, University of Washington, Seattle, USA

**Keywords:** Human cerebellar development, Non-coding genome, Gene regulatory networks, DNA methylomes, Histone modifications, Origins of pediatric hindbrain cancer

## Abstract

The cerebellar rhombic lip (RL) of the prenatal hindbrain is a progenitor niche essential for cerebellar glutamatergic neurogenesis. Humans demonstrate a structural elaboration of this niche with a rhombic lip subventricular zone (RL-SVZ). Disruption of this zone causes cerebellar malformations and tumors, yet its gene regulatory networks are poorly understood. We present a predicted gene regulatory network for the human RL inferred from epigenomic maps of the developing human cerebellum. We generated DNA methylomes from microdissected mid-gestation human RL ventricular zone (RL-VZ) and RL-SVZ (*N* = 9; 15–16 post-conception weeks) using low-input Enzymatic MethylSeq, and profiled histone marks of active promoters and enhancers in whole fetal cerebellum (*N* = 6; 14 and 18 weeks). Transition from RL-VZ to RL-SVZ is accompanied by widespread hypomethylation, including 9,855 differentially methylated regions (DMRs) enriched for binding sites of ATOH1, NEUROD1/2, and HMGA1. Of these, 88.9% are hypomethylated in RL-SVZ, enriched in active enhancers and human accelerated regions, and depleted at promoters. By integrating DMRs with bulk tissue histone maps as well as single-cell chromatin accessibility and transcriptomic maps of the mid-gestation cerebellum, we inferred over 100,000 transcription factor–enhancer–gene links. Twenty-five DMRs overlap human accelerated regions near genes implicated in intellectual disability, autism spectrum disorder, and neurological deficits. DMRs are also enriched in copy-number aberrations in medulloblastoma, with ~ 25% overlapping known aberrant regions, nominating disrupted promoters and enhancers. These data provide a framework for interpreting non-coding variation in human cerebellar development and disease.

**Supplementary Information:**

The online version contains supplementary material available at 10.1007/s12311-026-02057-4.

## Introduction

While the developing human cerebellum initially shares similarity with non-human primates and mice, important differences emerge in the first trimester; one such difference arises in the rhombic lip neurogenic niche [1–3]. This progenitor zone generates all glutamatergic neurons in the cerebellum, including granule neuron progenitors which produce granule neurons, which comprise over half of all neurons in the adult human brain [4], and unipolar brush cells. The human cerebellar rhombic lip has two anatomical compartments. The first is the SOX2^+^ KI67^+^ rhombic lip ventricular zone. The second is the SOX2^−^ KI67^+^ rhombic lip subventricular zone, which to date has only been seen in the developing human brain, but not mice or macaques [3, 5, 6]. In contrast, mice demonstrate four molecularly distinct developmental compartments in the cerebellar rhombic lip, with the main two compartments showing inverse expression of Pax6 and Wls [7]; the implications of the differences between rhombic lip compartmentalization in the two species is unknown to date. At age E15.5–17.5, which roughly corresponds to the mid-gestation period in humans, the mouse rhombic lip comprises of an inner rhombic lip at the ventricular surface, as well as a TBR2/EOMES^+^ intermediate zone [7]. Developmental dysregulation of the rhombic lip is hypothesized to cause some types of childhood hindbrain cancers such as Group 3 and 4 medulloblastoma [5, 8], as well as cerebellar structural birth defects, such as Dandy-Walker malformation [2]. The rhombic lip is therefore an anatomical structure relevant to cerebellar development, growth disorders, and potentially to human cerebellar expansion across primate evolution [3, 6].

Our goal is to identify gene regulatory networks - enhancers, transcription factors, and target genes - of the rhombic lip ventricular and subventricular zones. Elucidating this network will nominate master regulators of human cerebellar neurogenesis and evolutionary expansion, and identify gene expression programs that may be unique to the human-enriched rhombic lip subventricular zone (RL-SVZ). Annotating the epigenomic landscape of the developing rhombic lip will also allow inference of the impact of non-coding genetic variation in cerebellar disorders on the transcriptome, and ultimately, on cellular phenotype.

The cerebellar rhombic lip is a small region that can be readily identified based on its neuroanatomical location and cellular density, and readily captured using tissue microdissection combined with low-input genome profiling technologies [3]. Here we profiled these compartments by combining laser-capture microdissection of the rhombic lip ventricular and subventricular zones, with Enzymatic MethylSeq (EMseq [9]). EMseq is a sensitive enzyme-based DNA methylation assay that does not damage the tissue as the traditional bisulfite sequencing assay does, and it excels at producing unbiased DNA methylome coverage with low starting input amounts. To nominate *cis* regulatory DNA elements active in the rhombic lip, we integrated the rhombic lip DNA methylomes with histone maps of the whole human fetal cerebellum. We used ChIP-seq to map peaks of H3K27ac, which marks active enhancers, H3K4me3, which marks active promoters, and RNA Polymerase II, using mid-gestation whole human fetal cerebellum as input. While more predictive of active *cis* regulatory DNA elements [10–12] than DNA methylation, these assays require orders of magnitude more input DNA, making them currently infeasible to map small tissue compartments such as the rhombic lip. To infer target genes of regions demonstrating changes in DNA methylation in the differentiating rhombic lip, we integrated our epigenomic maps with predictions of enhancer-target gene associations [14], and chromatin co-accessibility data from single-cell transcriptomic and chromatin accessibility maps of the mid-gestation human cerebellum [13], we provide two sets of inferred gene regulatory networks. Roughly one-quarter of all epigenetically dynamic loci overlap structural variants in Group 3 and 4 medulloblastoma. Collectively the novel fetal epigenomic maps and target gene predictions represent a data-rich starting point to infer gene regulatory networks driving cerebellar neurodevelopment and ascertain the transcriptomic impact of noncoding variation in diseases affecting cerebellar growth.

## Methods

### Neuroanatomical Isolation of Mid-gestation Rhombic Lip Cell Compartments and Generation of DNA Methylomes

**Fig. 1:**
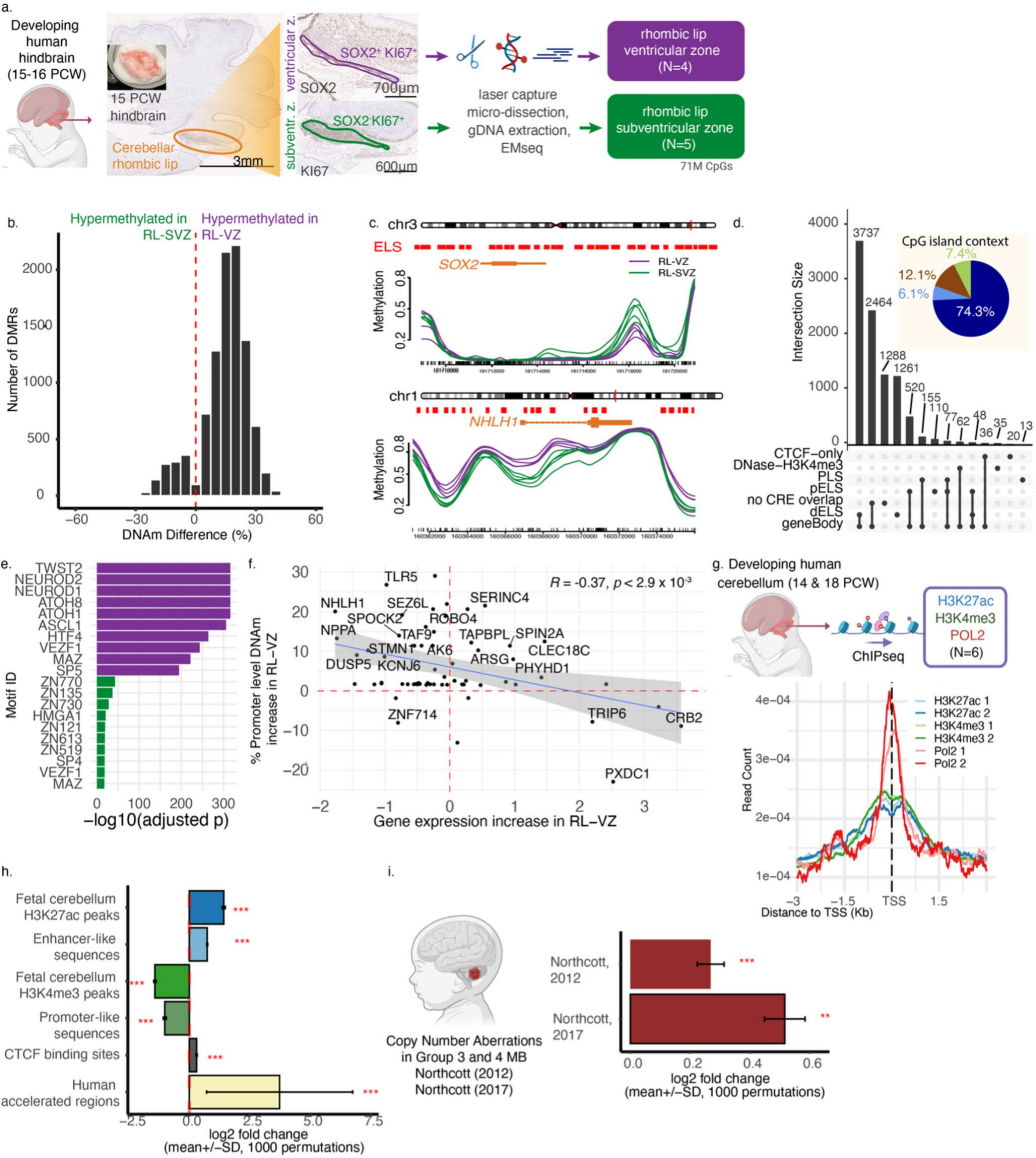
DNA methylome mapping of mid-gestation human rhombic lip reveals loci enriched in putative enhancers, human accelerated regions, and structural variants in medulloblastoma. (**a**) Workflow to generate DNA methylomes from mid-gestation human rhombic lip ventricular zone (RL-VZ) and rhombic lip subventricular zone (RL-SVZ). (**b**) Differentially methylated regions between RL-VZ and RL-SVZ (DSS, Q < 0.05). (**c**) Sample-level smoothed methylation around *SOX2*, a representative locus hypermethylated in RL-SVZ (top), and *NHLH1*, hypermethylated in RL-VZ. (**d**) Overlap of DMRs with ENCODE consensus *cis* regulatory elements and gene bodies. Inset shows fraction of DMRs overlapping CpG islands (green), CpG shores (brown), shelfs (light blue), and open sea regions (deep blue). “CTCF-only” indicates an ENCODE cCRE state that does not additionally overlap other CRE states. (**e**) Top ten most significant transcription factor binding site motifs for DMRs hypermethylated in RL-VZ (purple), and those for DMRs hypermethylated in RL-SVZ (green) (AME, Q < 0.05). (**f**) Change in promoter-level DNA methylation and corresponding gene expression in RL-VZ, versus RL-SVZ (*N* = 61 genes). Labelled genes are those which show an absolute DNA methylation change greater than 5% and also absolute RNA log fold-change greater than 0.1. (**g**) Schematic for generation of genome-wide chromatin immunoprecipitation data from human developing hindbrain (top) and average peak signal centered around transcription start sites (TSS). Each line indicates one technical replicate. (**h**) DMRs are enriched in enhancer-like regions, CTCF-binding sites, and human accelerated regions, and are depleted in promoter-like regions (permutation test, *p* < 10^− 3^). (**i**) DMRs are enriched in copy number aberrations in Group 3 and 4 medulloblastoma (permutation test, *p* < 10^− 3^). dELS: distal enhancer-like signatures; pELS: proximal enhancer-like signatures; PLS: promoter-like signatures; SE: super-enhancers

This project was approved by the Research Ethic Board at the University of Toronto (RIS Human Protocol #42524). We received frozen mid-gestation human hindbrains from the University of Maryland Brain and Tissue Bank (all males; mean age = 16 post-conception weeks, Supplementary Table 1). Informed consent was obtained from all donors. All methods were carried out in accordance with relevant guidelines and regulations laid out by the NIH NeuroBioBank, the U Maryland Brain and Tissue Bank, and the University of Toronto REB protocol. Tissue cryosectioning and immunohistochemistry was performed by the University Health Network Pathology Research Program Laboratory. Brain tissue was cryosectioned on the mid-sagittal plane and stained for cresyl violet, SOX2 (CST Sox2 (D6D9) XP Rabbit mAb #3579) and KI67 (Dako #M7240 Mouse monoclonal antibody). The rhombic lip was identified at the edge of the fourth ventricle by its size and prominent KI67 + staining (Fig. 1a, Supplementary Fig. 1). Selected sections were stained with CD34 (Dako #M7165 Monoclonal Mouse Anti-Human) and GFAP (Dako #Z334) to identify the vascular bed that separates the rhombic lip ventricular zone and subventricular zone (Supplementary Fig. 1). Using immunohistochemistry, we identified the rhombic lip ventricular (SOX2^+^ KI67^+^) and subventricular (SOX2^−^ KI67^+^) zone in each sample (Fig. 1a). We used laser capture microdissection (LCM) to isolate tissue separately from the rhombic lip ventricular zone and subventricular zone. LCM was performed by the Ontario Institute for Cancer Research Tissue Portal using the Leica LMD6 instrument; cresyl violet was used to visualize the tissue. We extracted nucleic acid from each tissue compartment, and generated DNA methylome profiles using the Enzymatic Methyl-seq assay (EM-seq; NEBNext Enzymatic Methyl-seq; Illumina NovaSeq 6000 and NovaSeq X; 150 bp paired-end reads; OICR Genomics Core) [15]. On average, 60.6 ng DNA was extracted for RL-VZ (range 7.1–115.8ng) and 350.3 ng DNA was extracted for RL-SVZ (range 115.8ng – 1,123.8ng) (Supplementary Table 1).

### EM-seq Data Analysis

#### Processing

A standard bioinformatics pipeline was used to process the EMseq data; processing statistics are reported in Supplementary Table 2. Fastp 0.23.2 [14] was used to ascertain read quality and perform adapter trimming. Reads were aligned to the hg38 genome using bwa-meth 0.2.5 [15]. Duplicates were filtered using Picard MarkDuplicates (https://broadinstitute.github.io/picard/*).* MethylDackel (https://github.com/dpryan79/MethylDackel) was used to compute Mbias plots. MethylDackel *extract --maxVariantFrac 0.2 --minOppositeDepth 5 --cytosine_report --OT 0*,*00147 --OB: 3*,*05*,*0* was used to compute cytosine-level methylation reports while removing potential C > T variants. Methylation non-conversion rate was measured using the methylation level of the lambda phage genome. Samples had a mean non-conversion rate of 0.21% (range 0.34–0.64) and all samples were retained. All samples showed consistent patterns of genome-wide methylation with over 50% of CpGs having 90% methylation and 10% CpGs with < 10% methylation (Supplementary Fig. 2). Hierarchical clustering of CpG methylation in the region of SOX2 and EOMES largely separated the rhombic lip ventricular zone and subventricular zone samples, and no samples were excluded from downstream analysis (Supplementary Fig. 3).

#### Differentially Methylated Regions

Differentially methylated regions (DMRs) were called for the CpG context using DSS [16]. Base-level hits were identified using *DMLtest*() with the following parameters: *smoothing=TRUE*,* smoothing.span = 500*. Base-level statistics were merged into region-level hits using *callDMR()* with parameters *delta = 0*,* p.threshold=1e-05*,* minlen = 50*,* minCG = 4*, *dis.merge = 100*,* pct.sig = 0.5*. We used AME for transcription factor binding site enrichment [17]. To test for enrichment of transcription factors active in the tissue of interest, we used the HOCOMOCO v12 CORE database as input after removing transcription factors expressed in less than 1% of rhombic lip ventricular or subventricular zone cells in a published snRNA-seq dataset [17].

#### Region Enrichment Test

To compute statistical enrichment of DMRs in regions of interest, we used a permutation test. The fraction of DMR overlap with target regions was compared to those from a length- and GC-matched set of genomic intervals sampled from mappable regions of the genome (*gkmSVM::genNullSeqs()*). Regions with < 90% overlap with Bismap-mappable regions of the genome [18] were excluded. This process was repeated 1,000 times, using different random sets of genomic intervals. The fraction of times the null statistic was greater than or equal to that from the real data, was reported as the p-value. In the CpG density matched version of this analysis, each set of negative sequences was matched to the CpG density of the positive sequences. For this, CpG density was counted in positive and negative sequences, and each negative sequence set was subsampled to match quartiles of CpG density in positive sequences. CpG density was defined as CpG dinucleotide frequency normalized by the length of the sequence.

#### Differentially Expressed Genes (DEG) in RL-VZ Versus RL-SVZ

Processed RNAseq data for neuroanatomically-dissected human rhombic lip ventricular zone and subventricular zone were downloaded from previously published work [6]. These data consist of RNA-seq from rhombic lip ventricular zone and rhombic lip subventricular zone, each extracted from a total of nine biological replicates. For the current work, analysis was limited to samples of age 15 PCW to 22 PCW (*N* = 6 samples, 3 per group) to match the developmental stage at which we generated DNA methylomes while maintaining a minimum of three samples per group to allow DEG inference. A standard bioinformatics pipeline and edgeR [19] was used to call differentially expressed genes; false discovery rate was controlled using the Benjamini-Hochberg approach. Genes with Q < 0.05 and with a non-zero log_2_ fold-change were deemed differentially expressed between the two compartments.

#### Gene Regulatory Network Inference

Predicted enhancer-gene links for H1-derived neural progenitor cells were downloaded from the companion website for Activity-by-Contact [20, 21] (ABC; see “External data and annotation sources” table below) and *liftOver* was used to map coordinates to hg38. DMRs were mapped to genes using these links. AME was used to infer transcription factor motifs overlapping a given DMR coordinate (ie., predicted enhancer) (union of *sequences.tsv* entries accompanying AME output for hypomethylated and hypermethylated DMRs, “true positive” sequences only). Where a DMR overlapped multiple TF binding sites, all were retained. Predicted TFs were filtered for those differentially expressed between the rhombic lip ventricular zone and subventricular zone [3] (genes with nominal p-value < 0.05). Gene set enrichment analysis of DMR target genes was performed using the *gProfiler2* R package [23], with the set of all ABC target genes as a custom background, and the following settings: *organism = “hsapiens”*,* significant = TRUE*,* sources = c(“GO: BP”*,* “GO: CC”*,* “REAC”*,* WP”*,* evcodes = TRUE*,* correction_method = “fdr”)*.

#### Association of Promoter-level DNA Methylation with Gene Expression

Promoter level DNA methylation was compared to corresponding transcription levels in two different ways. First, for each gene, promoter-level DNA methylation was computed as the average methylation over a region Xbp upstream and 1000 bp downstream of the TSS, where multiple values of X between 1000 bp and 5000 bp were tested. Only regions overlapping encode consensus promoter elements (chromatin states “PLS”, “PLS-CTCF-bound”) and those with a locus-level minimum coverage of 5 reads in every sample, were included. Locus-level methylation was averaged across samples within a neuroanatomical compartment, and the methylation increase in rhombic lip ventricular zone (RL-VZ) was computed. This value was compared against gene expression level for genes significantly upregulated in the RL-VZ.

Separately, we limited analysis to just rhombic lip DMRs, and compared these to log_2_ fold-change of transcription in the RL-VZ.

### Human Fetal Cerebellum ChIP-seq Data Processing and Peak Calling

Acquisition of human tissue samples was approved by the Seattle Children’s Hospital Institutional Review Board. Two fresh frozen specimens from male fetal (14 and 18 PCW) human cerebellum were obtained from the Birth Defects Research Laboratory at the University of Washington with ethics board approval and maternal written consent obtained before specimen collection. Fresh frozen cerebellum (130–180 mg) was sent to Active Motif for ChIP-seq using the following antibodies: H3K4me3 (Active Motif, cat#39159), H3K27Ac (Active Motif, cat #39133) and RNA Pol II (Active Motif, cat # 91151).

#### Sequence Analysis

The 75-nt single-end (SE75) sequence reads generated by Illumina sequencing (using NextSeq 500) are mapped to the genome using the BWA algorithm [22] (“bwa aln/samse” with default settings). Only reads that pass Illumina’s purity filter, align with no more than 2 mismatches, and map uniquely to the genome were used in the subsequent analysis. In addition, duplicate reads (“PCR duplicates”) were removed.

#### Determination of Fragment Density

Since the 5´-ends of the aligned reads (= “tags”) represent the end of ChIP/IP-fragments, the tags were extended in silico (using Active Motif software) at their 3´ ends to a length of 200 bp, which corresponds to the average fragment length in the size-selected library. To identify the density of fragments (extended tags) along the genome, the genome was divided into 32-nt bins and the number of fragments in each bin was determined.

#### Peak calling

Processing statistics are in Supplementary Table 3. Peaks were called using either MACS 2.1.0 [23] or SICER [24] algorithms. MACS default cutoff was pvalue 10^− 7^ for narrow peaks and 1e-1 for broad peaks. SICER default cutoff was FDR 10^− 10^ with gap parameter of 600 bp. Peak filtering was performed by removing false ChIP-Seq peaks as defined within the ENCODE blacklist [25].

Proximal H3K27ac peaks within 2 kbp upstream or downstream of transcription start sites that did not overlap with H3K4me3 peaks or distal H3K27ac peaks residing outside the +/−2k bp window were annotated as enhancer-like elements. H3K4me3 peaks overlapping transcription start site +/- 200 bp windows were annotated as canonical promoter-like elements [26]. The elements were annotated for each sample and then combined for downstream analyses.

Superenhancers were called by the ActiveMotif pipeline. The identification of Super Enhancers uses a proprietary algorithm that gives a very similar result as the ROSE software. In a first step, MACS or SICER peaks generated by the standard ChIP-Seq analysis are merged (or “stitched together”) if their inner distance is equal or less than 12,500 bp. In the second step, the stitched peak regions with the strongest signals (top 5%) are identified as Super Enhancers. Super Enhancer intervals are ranked by their signal strength (= tag numbers in stitched peak regions). For each sample, the top-ranked Super Enhancer is thus at the top of the list.

### Analysis of Single-Cell Chromatin Accessibility and Gene Expression Data from Mid-Gestation Cerebellum

This analysis used data generated by Sarropoulos et al. [26] Seurat and Signac [27] were used for downstream analysis. Processed human cerebellum snRNA-seq and snATAC-seq data were downloaded from https://apps.kaessmannlab.org/cerebellum_genreg_evodevo_app/. From the master set of cerebellar samples, samples of midgestation age were filtered (*Stage_group* variable = {“11wpc”, “15-17wpc”}; samples SA038, SA086, SA191, SA192, SA206, AND SA207, aged 11PCW to 16PCW), resulting in a total of 17,841 cells for the snATACseq data.

#### Inferring Enhancer-Promoter Pairs

In the ATAC-seq data, all peaks were confirmed to have blacklist ratios below 1.9 × 10^− 3^. Dimensionality reduction was performed using *RunTFIDF()*, *FindTopFeatures(min.cutoff = ‘q0’)*, and singular value decomposition. To identify co-accessible chromatin peaks in the rhombic lip, the ATAC data was subset to only include cells with *rna_precisest_label = “progenitor_RL”* (246 cells). Cicero [28] was run on chromosomes 1–22, X and Y, to call co-accessible peaks. Only peaks with a co-accessibility score greater than 0.25 were retained (6,250,506 peaks). To identify high-confidence enhancer-promoter pairs (“inferred EP pairs”), Cicero co-accessbility peak pairs were filtered so that exactly one of the peaks in the pairs overlapped a gene transcription start site (13,151 peaks mapping to 292 genes). For this, Gencode v44 basic annotation was used to define gene extents [29, 30].

In the RNA-seq data, as with the ATAC data, cells belonging to Stage_group = {“11wpc”, “15-17wpc”} were filtered. This resulted in 17,841 cells in the ATACseq layer and 32,186 cells in the RNAseq layer. *SCTransform()* was used to normalize gene expression values, adjusting for cell cycle phase differences. Dimensionality reduction was performing using PCA, and UMAP visualization was used to confirm author-assigned cell labels existed in the same cluster. To identify differentially expressed genes in the rhombic lip, *FindMarkers()* was run in one-versus-all format, with the settings *min.pct = 0.1*,* test.use = “wilcox”*. Genes with Q < 0.1 and average log_2_ fold-change > 0 were deemed to be upregulated in rhombic lip cells.

#### Changes Through Rhombic Lip Differentiation Stages

The precisest level of cell state/subtype annotation assigned by Sarropoulos et al. was used to label cell states here (ATAC: *“rna_precisest label”* metadata column, RNA: *“precisest_label”* metadata column). Global changes in chromatin accessibility and gene expression for different cell stages were inferred by applying Seurat’s *FindMarkers()* to pairs of cell states. For both layers, FindMarkers was used with settings *min.pct = 0.1*,* test.use = “wilcox”*. For the RNA layer, the SCT assay was used for differential gene expression testing. Peaks or genes with adjusted p-value < 0.05 were deemed significant. Only peaks or genes showing significant differences between the groups were used for directionality analyses.

#### Gene Regulatory Network Inference

For gene regulatory network (GRN) inference, only DMRs that overlapped inferred EP pairs were retained (these are called “eDMRs”). To identify significantly-occurring transcription factor binding site motifs in individual DMR sequences, we used a position weight matrix based scoring approach (FIMO [31]). Cytoscape 3.9.0 was used to visualize the GRN subnetwork associated with neurodevelopmental or medulloblastoma driver genes [32].

#### Association of DMRs with Copy Number Aberrations

GISTIC [33]-called copy number amplifications and deletions (Copy Number Aberrations or CNAs) in Group 3 and 4 medulloblastoma were obtained from previous studies [34, 35. Only protein-coding genes were used for ascertaining gene overlap with DMRs/eDMRs and CNAs; for this, Gencode v44 basic annotation was used to define gene extents.

**Table:** External data and annotation sources

CpG islands, shores, shelves and open sea	*R* package annotatr [36].
Gene definitions	GENCODE [34] https://ftp.ebi.ac.uk/pub/databases/gencode/Gencode_human/release_42/gencode.v42.basic.annotation.gtf.gz For some analyses, v44 was used.
“Neurodevelopment-associated genes”: Marker genes associated with neuronal subtypes in human or murine cerebral cortex or hindbrain development.	Refs. [1, 3, 37–40].
Group 3 and 4 medulloblastoma-associated genes	Refs. [5, 35].
Predicted Human Accelerated Regions [41]	https://docpollard.org/wordpress/wp-content/research/nchaes_merged_hg19.bed. LiftOver used to map coordinates to hg38.
ENCODE consensus cis-regulatory elements	https://downloads.wenglab.org/V3/GRCh38-cCREs.bed
Copy number aberrations in Group 3 and 4 medulloblastoma	GISTIC [33]-called amplifications and deletions from refs. [34, 35]
Activity-by-Contact predicted enhancer-gene links for H1-derived neural progenitors [37, 38]	https://mitra.stanford.edu/engreitz/oak/public/Nasser2021/AllPredictions.AvgHiC.ABC0.015.minus150.ForABCPaperV3.txt.gz.

## Results

### DNA is Predominantly Hypomethylated in Putative Enhancers As Cells Transition From the Rhombic Lip Ventricular Zone to Subventricular Zone, Converging on Binding Sites of Known Regulators of Rhombic Lip Differentiation

We generated DNA methylomes for mid-gestation human rhombic lip ventricular (RL-VZ) and subventricular zones (RL-SVZ) (Fig. 1a, *N* = 9 samples from 5 males; 15–16 post-conception weeks; Supplementary Tables 1–2, Supplementary Fig. 1–3) and looked for regions showing epigenetic dynamics over the course of rhombic lip differentiation. We identified 9,855 regions that showed significant differential CpG methylation (DMR) between the RL-VZ and RL-SVZ (DSS [16]). The vast majority of DMRs were hypermethylated in the RL-VZ (88.5%), and were of an average length of 360 bp (Fig. 1b-c; Supplementary Fig. 4, Supplementary Table 4; range of 51 − 4,122 bp length; mean = 46.3% hypomethylation). Figure 1c shows detailed views of sample-level methylation of DMRs in the genomic regions around *SOX2* and *NHLH1*, two genes known to regulate neurodifferentiation. Only 7.4% of DMRs overlapped CpG islands, and similar fractions overlapped CpG shores (12.1%) and shelves (6.1%); most DMRs were located outside these regions (74%; “open sea”) (Fig. 1d). Over 80% of DMRs overlapped enhancer-like sequences, with the vast majority overlapping distal enhancer-like sequences [26] (72.4%; 10.6% overlap proximal enhancer-like sequences; Supplementary Table 5). In contrast only 2.8% of DMRs overlapped promoters (Fig. 1d).

DMRs hypomethylated in the RL-SVZ were enriched for binding sites for 339 transcription factors, with the top three being NEUROD1, NEUROD2, ATOH1 (Fig. 1e; AME, Q < 0.05; Supplementary Table 6). These transcription factors are known master regulators of rhombic lip cell identity and neuronal differentiation, with ATOH1 being an established regulator of glutamatergic cell identity in the rhombic lip [1]. In contrast, DMRs hypomethylated in the RL-VZ are enriched for 159 transcription factor motifs, including HMGA1 and ZN519 (Fig. 1e, AME Q < 0.05; Supplementary Table 7).

We compared promoter-level DNA methylation change to change in gene expression, using a previously published transcriptome of micro-dissected human RL-VZ and RL-SVZ (15–22 PCW, *N* = 6) [3]. The direction of promoter-level methylation change of DMRs is negatively correlated with that of gene expression change (Fig. 1f, *N* = 61 loci, Pearson rho = −0.37, *p* < 2.9 × 10^− 3^, promoter region defined as 2000 bp upstream of TSS 10 1000 bp downstream of TSS; data for different extents of promoter shown in Supplementary Fig. 5a) (Supplementary Table 8). Globally, promoter-level DNA methylation demonstrates a weak but significant negative correlation with transcription (Supplementary Fig. 5b, *N* = 2,005 genes; Pearson rho = −0.09, *p* = 5.6 × 10^− 5^).

To focus on the context of the developing cerebellum, we generated maps of histone modifications for active enhancers (H3K27ac), promoters (H3K4me3), and transcription (POL2) from the bulk fetal cerebellum (Fig. 1g, *N* = 2 biological replicates, male, 14 and 18 post-conception weeks; Supplementary Table 3). We found that just under one-third of DMRs overlapped a putative enhancer or promoter region as defined by peaks of histone modifications in this tissue (31.1%, or 3,061 DMRs) (Supplementary Table 9). Most of these overlapped with peaks of active enhancer marks (24.4% of all DMRs), with only 8% overlapping putative promoters. Indeed, we found that DMRs were enriched in H3K27ac peaks in the fetal cerebellum, relative to length, GC- and mappability-matched sequences (Fig. 1h, *p* < 0.001). Importantly, DMRs were statistically depleted in H3K4me3 peaks in the human fetal cerebellum, a mark for active promoters (*p* < 0.001). We obtained the identical result using ENCODE CRE definitions of enhancers and promoters [26] (*p* < 0.001). The fetal cerebellar H3K27ac maps revealed a set of 986 superenhancers (median length=26 kb; range = 2.8 kb to 191 kb; Supplementary Table 10). The nearest genes to these superenhancers included 28 genes previously linked to cerebellar neurodevelopment, including *NFIA*, *OTX2*, *EOMES*, *LMX1A*, and *ZIC1* (median of control sequences = 1 gene overlap, *p* < 1 × 10^− 3^, permutation test). Notably, 36 of the superenhancers had known Group 3 and 4 medulloblastoma drivers as their nearest genes, including *CHD7*, *CBFA2T2*, *GSE1*, *OTX2*, and *ZIC1* (median of control sequences = 1 gene overlap; *p* < 1 × 10^− 3^; permutation test). Notably, 1,130 DMRs (11%) overlap regions predicted to contain superenhancers in the fetal cerebellum (4.63% median overlap of control sequences; *p* < 1 × 10^− 3^, permutation test); almost all of these were hypomethylated in the RL-SVZ (1,028 DMRs, or 91.0%) (Supplementary Table 11).

We next looked at DMR overlap with regions of accelerated evolution in the human genome, and with DNA copy number aberrations in Group 3 and 4 medulloblastoma. We found that 25 DMRs overlap human accelerated regions [41] (enrichment; 0.25% DMRs, 0.03% median overlap of control sequences, *p*<1 × 10^− 3^, permutation test; Fig. 1h, Supplementary Table 12); all but one of these are hypomethylated in the RL-SVZ. While the CpG density of DMRs is higher than those of negative sequences, DMRs continue to be enriched in superenhancers relative to negative sequences even after matching quartiles of CpG density (*p*<1 × 10^− 3^, permutation test; Supplementary Fig. 6). These include regions with nearest protein-coding genes such as *BCAS3*, *RFX3*, *SOBP*, and *PEX14*, mutations in which have been linked to intellectual disability, neurological deficits, attention deficit hyperactivity disorder, or autism spectrum disorders [42–45].

To examine enrichment of DMRs in copy number aberrations, we considered copy number aberrations (CNA) from two independent publications and called using two different genomic platforms [34, 35. We found that DMRs were enriched in both sets of CNA peaks tested (Fig. 1i, *p* < 1 × 10^− 3^ in both instances). Close to one-quarter of the DMRs overlap known amplifications and deletions in Group 3 and 4 medulloblastoma (*N* = 2,135 DMRs, or 22%). The complete list of DMRs annotated with overlapping target genes and tumour CNAs is provided in Supplementary Table 13. Rhombic lip DMRs located in copy number aberrations may point to *cis*-regulatory DNA elements that may be amplified or deleted in medulloblastoma, resulting in a functional change in gene regulation, and in driving the cancer. We find that DMRs are enriched in copy number amplifications and deletions found in both Group 3 and Group 4 MB genomes (*p*<1 × 10^− 3^, all instances, Supplementary Fig. 7a, Supplementary Table 13). An average of 679.2 DMRs overlap CNAs in each category, with 458 DMRs overlapping Group 3 MB amplifications (4.6%), 477 DMRs overlapping Group 4 MB amplifications (4.8%), 913 DMRs overlapping Group 3 MB deletions (9.3%), and 869 DMRs overlapping Group 4 MB deletions (8.8%) DMRs overlapping CNAs also overlap a mean of 178.2 genes in each of these four categories (Supplementary Table 14; Supplementary Fig. 7b). In all instances, the DMRs are predominantly hypomethylated in the RL-SVZ (87.9% − 90.1% hypomethylated).

Collectively, our data point to widespread DNA demethylation as cells transition from the RL-VZ to the RL-SVZ state, with epigenetic changes converging on binding sites transcription factors promoting neuronal differentiation. This hypomethylation appears to be enriched in enhancers – rather than promoters –, and in regions showing accelerated evolution in humans. Notably, regions showing this epigenetic change are enriched for loci that are amplified or deleted in Group 3 and 4 medulloblastoma. We next used these data as a starting point to narrow down individual candidate regions as high-confidence enhancer predictions.

### Predicted Cerebellar Rhombic Lip Gene Regulatory Network Identifies New Transcription Factor Links to Developmental Genes, Medulloblastoma Driver Genes, and CNAs

**Fig. 2:**
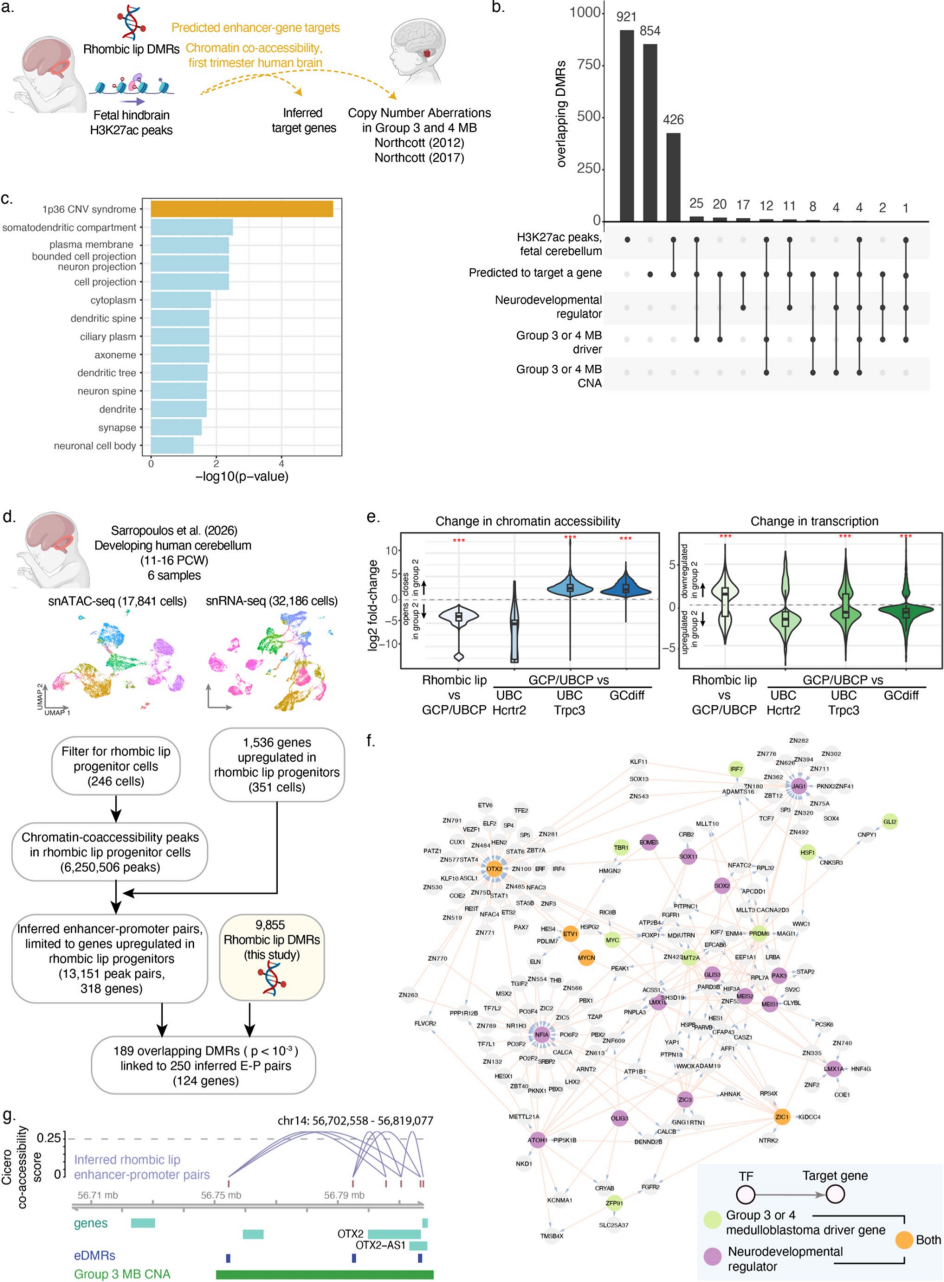
Annotation of differentially methylated regions with inferred target genes and medulloblastoma structural variants. **a**. Schematic for annotation. **b**. Overlap of putative rhombic lip candidate enhancers with fetal cerebellum active enhancer marks, cortical neural progenitor active enhancer marks and genetic aberrations in Group 3 and 4 medulloblastoma. Also annotated are regions where the nearest or predicted target gene is a known regulator of neurodevelopment or Group 3 and 4 medulloblastoma driver gene. Top five categories shown. **c**. Gene set enrichment of DMR target genes; FDR-adjusted p-values shown. WikiPathways entry shown in orange; GO Cellular Compartment entries shown in blue. **d**. Schematic for inferring enhancer-gene pairs for human rhombic lip progenitor cells. **e**. Direction of changes in chromatin accessibility (left) and altered genes (right) in pairwise comparisons of dividing and differentiating cell states of the rhombic lip lineage. Only significantly different peaks/genes are included in the analysis. Asterisks from one-tailed t-test. Chromatin accessibility: *N* = 246 cells, rhombic lip, *N* = 131 cells, GCP/UBCP; *N* = 48 cells, UBC Hcrtr2; *N* = 613 cells, UBC Trpc3; *N* = 3,027 cells, GCdiff. Gene expression: *N* = 351 cells, rhombic lip, *N* = 728 cells, GCP/UBCP; *N* = 158 cells, UBC Hcrtr2; *N* = 1,113 cells, UBC Trpc3; *N* = 3,866 cells, GCdiff. **f**. Predicted gene regulatory network in the human rhombic lip where DMRs overlap inferred enhancers. Source nodes indicate predicted transcription factor binding sites overlapping inferred enhancers. Only links associated with genes of neurodevelopmental (purple) or Group 3 and 4 medulloblastoma (yellow-green) relevance and also in the top decile of co-accessibility scores are shown in this subnetwork (Supplementary Table 16). **g**. View of enhancer DMRs (eDMRs) overlapping a Group 3 MB copy number amplification at the *OTX2* locus. GCP/UBCP: granule cell/unipolar brush cell progenitors; UBC Hcrtr2, UBC Trpc3: differentiating UBCs. GCdiff: Differentiating granule cells

We next sought to analyze the DMRs to nominate a list of predicted enhancers and their target genes. For this purpose, we integrated the rhombic lip specific DMRs with the fetal cerebellar histone maps generated above, with previously predicted enhancer-gene pairs from multimodal epigenomic data [49], and with chromatin co-accessibility data from the mid-gestation cerebellum^50^ (Fig. 2). To explore the hypothesis that medulloblastoma CNAs disrupt developmental enhancers, we also examined the overlap between DMRs, predicted enhancers, and CNAs.

14% of DMRs overlap active enhancer marks, as defined by H3K27ac peaks in the fetal cerebellum (Fig. 2a-b, *N* = 1,400 DMRs). As our interest is in identifying non-coding DNA elements that impact the regulation of genes involved in neurodifferentiation and medulloblastoma oncogenesis, we mapped DMRs to genes using enhancer-gene predictions from chromatin accessibility and DNAseI hypersensitivity data [51]. Separately, 14% of DMRs are predicted to regulate the expression of 4,604 genes (*N* = 1,384 DMRs, Supplementary Table 9) [52]. Rhombic lip DMR target genes were enriched for genes associated with neuronal cell compartments, including dendritic spines and synapses (Fig. 2c). Target genes were also enriched in genes associated with 1p36 Copy Number Variation syndrome, a genetic congenital disorder arising from 1p36 deletions, and associated with developmental delay, intellectual disability, and structural anomalies of the brain, among other symptoms [46]. DMR target genes were slightly enriched in genes differentially expressed in mid-gestation RL-VZ versus RL-SVZ [6] (16.9%, relative to 15.5% of background genes; Fisher’s Exact Test, *p* < 0.02). Collectively, DMR target genes appear to be enriched in genes expressed in specialized cellular compartments of neurons.

We predicted transcription factor-enhancer-target gene links (triplet links) using rhombic lip DMRs and annotating these with both, overlapping transcription factor motifs and predicted target genes [10, 17] (Supplementary Table 15). To identify triplet links, we limited our transcription factor set to genes previously shown to be differentially expressed between the rhombic lip ventricular zone and subventricular zone [3]. We identified a total of 81,844 triplet links, covering over 98% of DMRs (9,741 DMRs). The links are associated with 41 transcription factors and include 4,610 predicted target genes. 29 DMR target genes are those previously associated with neurodevelopmental regulation, including *SOX2*, *WLS*, *PAX6*, and *MKI67* (Supplementary Table 15) (*p* < 1 × 10^− 3^, permutation test using length-, GC- and mappability-matched sequences). Our network includes previously known gene regulatory connections such as the regulation of *Notch1* and *Otx2* by Sox2 [47, 48], and regulation of *PAX6* by SMAD3^49^. DMRs were also predicted to regulate the RUNX1 transcriptional co-repressors *CBFA2T2* and *CBFA2T3*, which have been shown demarcate the rhombic lip subventricular zone. *CBFA2T2* and *CBFA2T3* are recurrently mutated in Group 4 medulloblastoma, a group of tumours hypothesized to originate in the rhombic lip subventricular zone [50]. Our triplet links also include 60 genes known to be recurrently mutated or overexpressed in medulloblastoma, including *OTX2*, *MYCN*, the Fanconi anemia proteins *FANCA* and *FANCI*, and others (Supplementary Table 15) (*p* < 1 × 10^− 3^, permutation test).

We next analyzed single-cell transcriptomic and chromatin accessibility data from the mid-gestation cerebellum [13] to recapitulate our findings and put the DMRs in the context of tissue-specific gene regulatory networks (Fig. 2d-g). As we had observed widespread DNA hypomethylation as cells transition from the RL-VZ to the RL-SVZ, we decided to compare this observation to the overall direction of chromatin accessibility change through rhombic lip differentiation. We observed that as cells transition from rhombic lip progenitors to granule cell progenitors/UBC progenitors (GCP/UBCP), there is an overall increase in chromatin accessibility (Fig. 2e, *N* = 246 rhombic lip cells, *N* = 131 GCP/UBCP cells; 3,133 peak changes with Q < 0.05, average log_2_ fold-change = −4.3; *p* < 2 × 10^− 16^, one-tailed t-test). In contrast, there is an overall decrease in chromatin accessibility as cells transition from GCP/UBCP to differentiating granule cells or UBC (Fig. 2e, *p* < 2 × 10^− 16^, one-tailed t-test). This does not translate to a direct correspondence with gene expression changes. A similar examination of the direction of differentially expressed genes reveals that differentially expressed genes tended to initially be downregulated, followed by a predominant upregulation of genes later in rhombic lip differentiation (Fig. 2e, one-tailed t-test, *p* < 10^− 8^ in all instances).

To more directly compare DMRs to enhancers of the developing rhombic lip, we separately inferred enhancer-gene pairs from the developmental single-cell resolution data analyzed above [51]. Roughly half the DMRs (*N* = 4,491 DMRs, 45.6%) overlapped the full set of rhombic lip chromatin co-accessibility peaks (6,250,506 peaks; *p* < 1 × 10^− 3^, permutation test). From the full set of chromatin co-accessibility pairs, we identified 13,151 inferred enhancer-promoter pairs that are associated with 318 genes upregulated in the developing rhombic lip. DMRs are also enriched in this set, with 189 DMRs overlapping 250 unique enhancers that target 124 unique genes (eDMRs; *p*<1 × 10^− 3^, median control sequences overlapping inferred enhancers = 69.5; Supplementary Table 16). Of these genes, 4 were neurodevelopment-related (*NFIA*, *OTX2*, *JAG1*, *LMX1A*), and only one was a known Group 3 and 4 medulloblastoma driver gene (OTX2) (overlaps not significant, *p* > 0.1, permutation test). As before, we inferred transcription factor-enhancer target gene triplet links using these rhombic lip-derived enhancer-promoter pairs (Fig. 2f, Supplementary Table 16). Using this approach, we identified 49,360 transcription factor-enhancer-gene links that included 598 unique transcription factors and 124 genes. This alternate approach generates a complementary set of hypotheses around gene regulatory networks active in the rhombic lip.

To nominate CNAs that potentially disrupt rhombic lip enhancers associated with particular genes, we examined the overlap of 189 eDMRs inferred above with CNAs. An average of 9 eDMRs overlapped CNAs; 8 eDMRs for Group 3 MB amplifications, 7 eDMRs for Group 4 MB amplifications, 15 eDMRs for Group 3 MB deletions, and 10 eDMRs for Group 4 MB deletions (*p* > 0.1, in all instances). These 28 eDMRs that overlap CNAs are connected to 16 unique genes (Supplementary Table 17). Notably, three eDMRs overlap a Group 3 MB CNA at the OTX2 locus; these eDMRs are predicted to regulate OTX2 (Fig. 2g). Two of these eDMRs lie downstream of the OTX2 transcription end site, and one lies in the vicinity of the transcription start site of the longest OTX2 transcript. Collectively, the DMRs highlight potential rhombic lip enhancers, which – when disrupted by copy number changes of the type seen in Group 3 and 4 MB tumours – have the potential to disrupt gene regulation.

## Discussion

We have mapped, to our knowledge, the first DNA methylomes of the neuroanatomically-dissected human cerebellar rhombic lip, the progenitor niche which goes on to generate granule cells that comprise over 80% of the neurons in the adult human brain [4, 50, 51], and the dysregulation of which is hypothesized to result in structural cerebellar birth defects and medulloblastoma. The advantage of our approach is that, unlike the cell type inference required from single-cell genomics datasets, here we directly sample neuroanatomical compartments of the rhombic lip ventricular and subventricular zones. From these data, we infer gene regulatory networks that include 41 transcription factors active in the rhombic lip, and target genes that mediate neurodevelopment and are drivers of Group 3 and 4 medulloblastoma. By integrating methylome maps with novel histone maps of the human fetal cerebellum that mark active enhancers and promoters, we predicted nearly 1,400 enhancers that regulate over 4,600 genes. These include enhancers of regulators of genes that drive glutamatergic neuronal identity (e.g., PAX6, WLS), and known drivers of medulloblastoma (OTX2, MYCN, CBFA2T2). The developmental timepoints used for both datasets are comparable, as in both cases, the fetal cerebellum experiences RL and EGL-driven cerebellar neurogenesis and expansion [51]. These data provide a rich starting point for functional studies to dissect the gene regulatory impact of non-coding variation observed in genomes of medulloblastoma tumours and patients with structural birth defects of the cerebellum.

To our knowledge, ours is the first report of widespread CpG hypomethylation as cerebellar glutamatergic neural progenitors transition from the RL-VZ to the RL-SVZ cell state (Fig. 1b). This hypomethylation converges on transcription factors that promote neurodifferentiation in this context, including NEUROD1 and NEUROD2 (Fig. 1e). A similar widespread CpG hypomethylation has been reported in the embryonic mouse cortex [52], where a transition from Nes^+^ NPCs to Dcx^+^ early differentiating neurons is accompanied by genome-wide CpG hypomethylation associated with binding sites of NeuroD2. Consistent with a more permissive chromatin state, we observed a global increase in chromatin accessibility as cells transition from rhombic lip progenitors to granule cell/UBC progenitors using single-cell genomic data (Fig. 2e). A similar increase in chromatin accessibility with neuronal differentiation has been documented in a recent single-cell multiomic study of the first trimester human brain [53]. Interestingly, in the cerebellum single-cell ATACseq data we analyzed, this trend appears to reverse through subsequent differentiated cell states. In an apparent contradiction, the global directionality of gene expression changes is the opposite to that seen in chromatin accessibility changes. The reason for this discrepancy is not apparent. One possible reason is chromatin priming [54], where changes in chromatin accessibility precede those of gene expression during lineage specification. Another possible reason includes the binding of repressive transcription factors which downregulate gene expression. In sum, our analyses suggest that chromatin accessibility and DNA hypomethylation increase in the early stages of rhombic lip progenitor stages. We note that while these findings will need to be validated with large single-cell multiomic or DNA methylomic datasets, the patterns are consistent with each other. Separately, we observed that binding sites for High Mobility Group AT-hook 1 (HMGA1) are enriched in regions hypomethylated in the RL-VZ. HMGA1 is a non-histone chromatin remodeller highly expressed in human and mouse stem cells, and whose levels are reduced in adult tissues, but which is reactivated in multiple cancers [55–58]. HMGA1 is associated with an activation of stem cell transcriptional networks, as observed in poorly-differentiated cancers [56], and overexpression promotes cardiomyocyte regeneration [59] and tumourigenesis [60]. HMGA1 enrichment is consistent with the activation of a stem cell program in the SOX2 + RL-VZ compartment. Our findings that compartment-specific DMRs are enriched overall in putative enhancer elements rather than promoters is consistent with the observation from the Roadmap Epigenomics project and others, that enhancer-like chromatin states display tissue specificity, whereas active promoters were constitutive [12]. Collectively, the epigenetic reprogramming reflected in these data are consistent with the gene regulatory changes known to accompany neurogenesis in general, and cerebellar neurogenesis in particular.

We note that a substantial fraction of DMRs did not overlap enhancers in the fetal ChIPseq dataset, nor in the consensus ENCODE cis-regulatory element set (Supplementary Table 9). One reason for this lack of mapping could be due to cell type mismatch between the two reference sets used for enhancer definitions. The fetal ChIPseq data generated here were extracted from whole bulk cerebellum, the majority of which at 17–18 PCW comprises of Purkinje cells and granule cells of the external granule cell layer. Consequently, despite being extracted from first trimester fetal hindbrain tissue, as the rhombic lip samples were, the enhancers from the ChIPseq data likely predominantly reflect those from non-rhombic lip cell types. Integration of these DMRs with cell type-specific enhancer maps, such as those generated from single-cell multiome data, may resolve compartment-specific enhancers. We cannot exclude the possibility that a fraction of the DMRs is specific to the developmental stage we sample here at 15–16 PCW; studies that longitudinally map gene regulatory networks in the developing cerebellum will be needed to distinguish persistent DMRs from stage-specific ones.

One limitation of the current study is that we used only male samples to generate DNA methylomes. Future studies will need to include female samples to ascertain if sex-specific epigenomic differences can explain the bias in incidence of some subtypes of medulloblastoma in males [61]. Another limitation of the current study is that we integrate DMRs from the neuroanatomically distinct and small glutamatergic rhombic lip, with histone marks from the whole fetal cerebellum. However, a large proportion of the latter includes GABAergic cells such as the Purkinje cell lineage, glutamatergic neurons in later states of differentiation (granule cells), and non-neuronal cell populations [1, 37]. As technologies for low-input histone mapping become more widely used, our predictions can be refined using annotations that are more specific to rhombic lip cells. Our inference of gene-regulatory networks may also suffer bias from the use of H1-derived neural progenitor cells, rather than rhombic lip cells. Finally, we note that within the respective compartments sampled here, our technique of laser-capture microdissection still results in a mixture of cells, likely including immature, differentiating granule cell progenitors and unipolar brush cells amid progenitor cells. A true definitive separation of these cell types may require the use of a genome-wide spatial assay.

Functional validation of disease variants predicted to impact gene expression will be necessary for eventual use of this information in genomic diagnostics [62, 63].

## Supplementary Information

Below is the link to the electronic supplementary material.

Supplementary Material 1 (XLSX 21.9 MB)

Supplementary Material 2 (DOCX 3.92 MB)

## Data Availability

Rhombic lip EM-seq data generated for this project have been deposited at Gene Expression Omnibus and are available under accession GSE313278. Fetal cerebellum H3K7ac, H3K4me and POL2 ChIPseq data are available under accession GSE313877. Called DMRs have been made available in supplementary data associated with this manuscript. Software to reproduce analysis in this manuscript is available at [https://github.com/RealPaiLab/RhombicLip_Epigenome_CLEAN](https:/github.com/RealPaiLab/RhombicLip_Epigenome_CLEAN) and will be made available under a Creative Commons Attributions License upon publication.
