# Supplementary material for: Differentially Methylated Regions in Human Rhombic Lip Compartments Are Enriched in Putative Active Enhancers, Human Accelerated Regions, and Medulloblastoma Copy Number Aberrations
Source: Cerebellum. 2026 Jul 21;25(4):114. doi: 10.1007/s12311-026-02057-4 (PMC13388357; doi:10.1007/s12311-026-02057-4)
Supplement: Supplementary file 2 — Supplementary Material 2 (DOCX 3.92 MB) [file 12311_2026_2057_MOESM2_ESM.docx]

**Supplementary Figures**


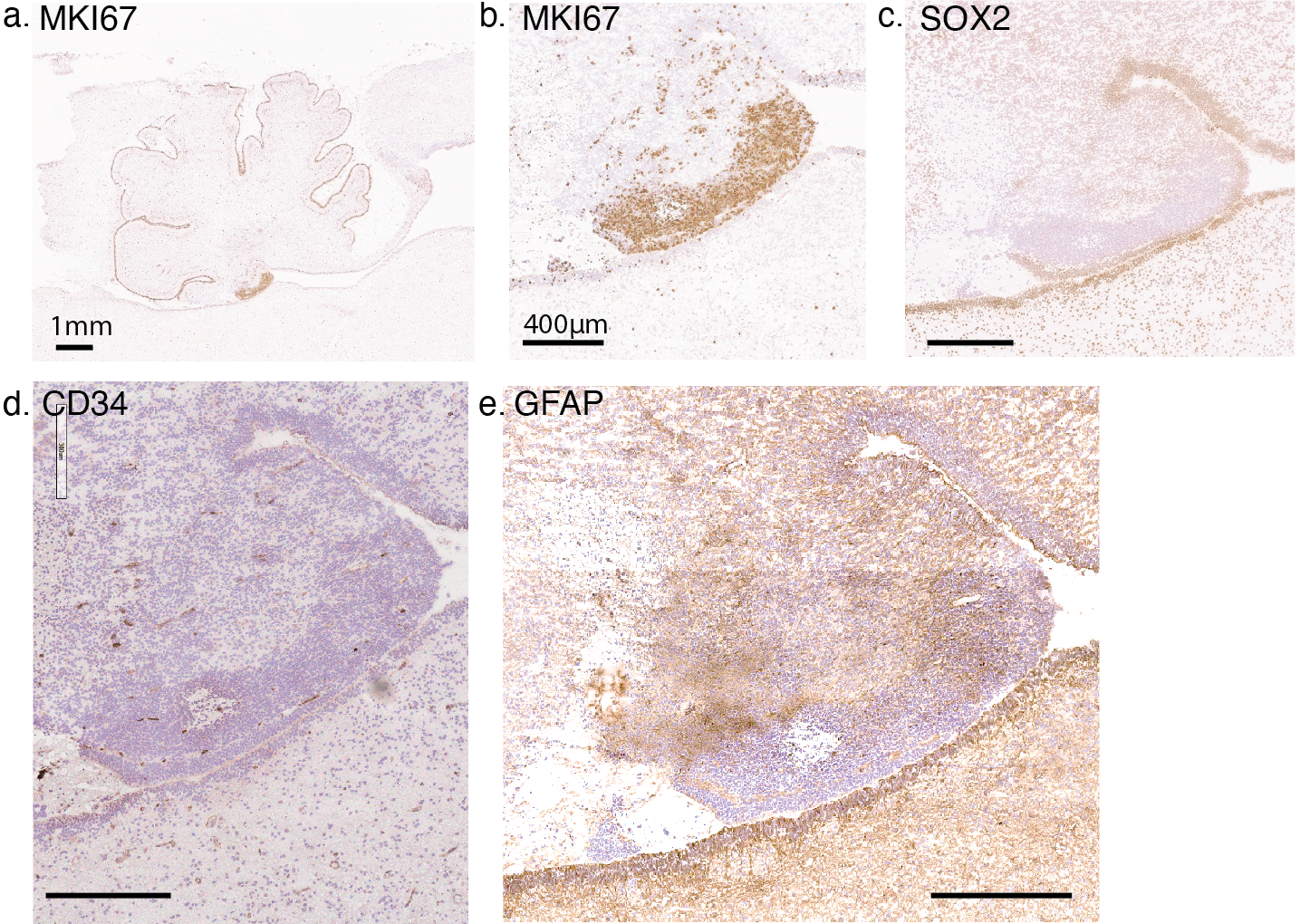


**Supplementary Fig. 1.** Immunohistochemistry of mid-sagittal fetal rhombic lip demarcating the rhombic lip ventricular zone (RL-VZ) and subventricular zone (RL-SVZ) regions, taken from a male aged 16 post-conception weeks. a. MKI67, a marker of cell proliferation, marking both the RL-VZ and the RL-SVZ. b. Closer view of the sample in panel a. c. SOX2, a marker of stemness, and one which marks the RL-VZ but not the RL-SVZ. d. CD34 and e. GFAP, which mark the vascular bed that separates the RL-VZ and RL-SVZ.


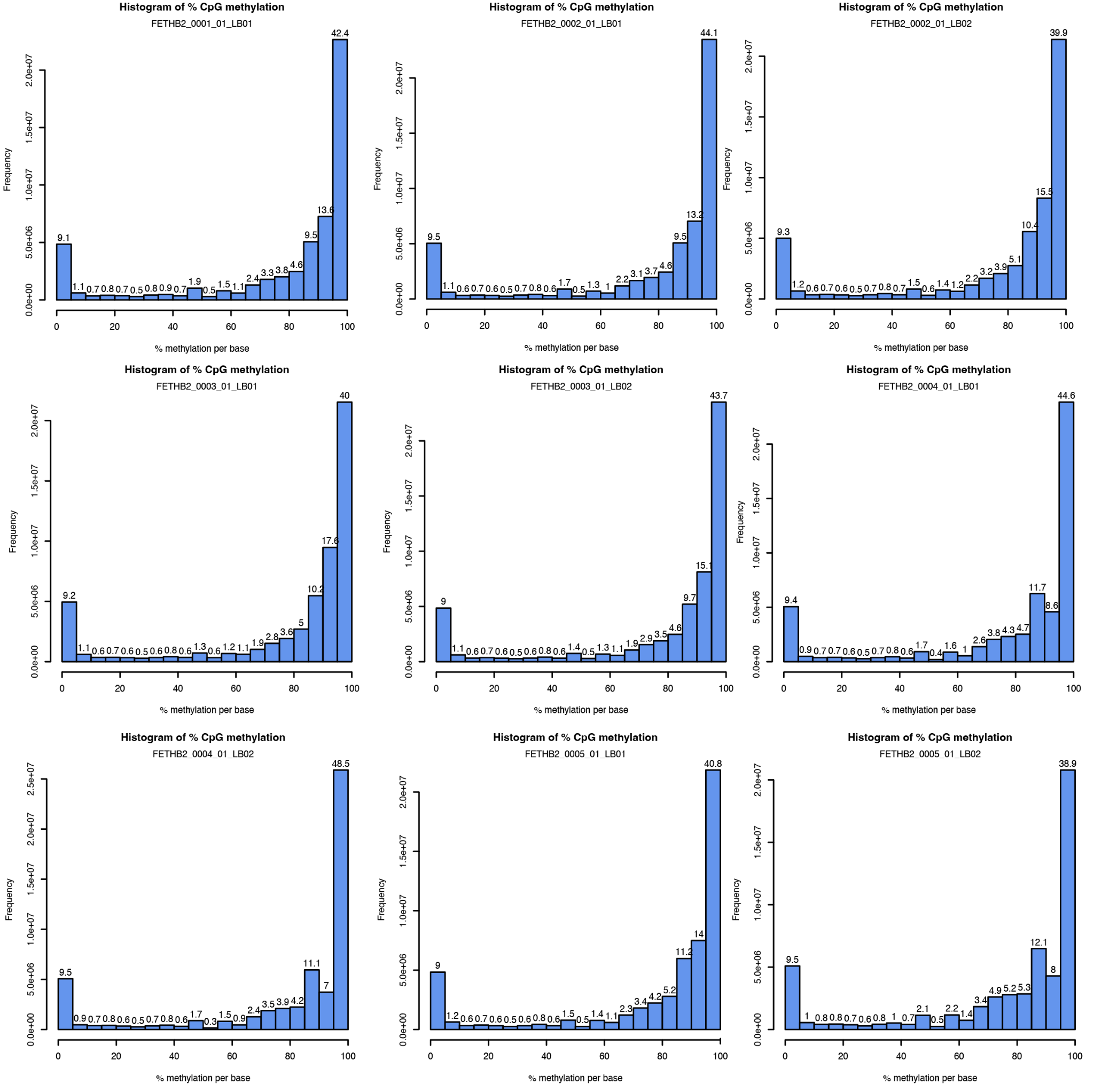


**Supplementary Fig. 2.** Genome-wide distribution of base-level percent DNA methylation. Each panel shows data for an individual DNA methylome.

**
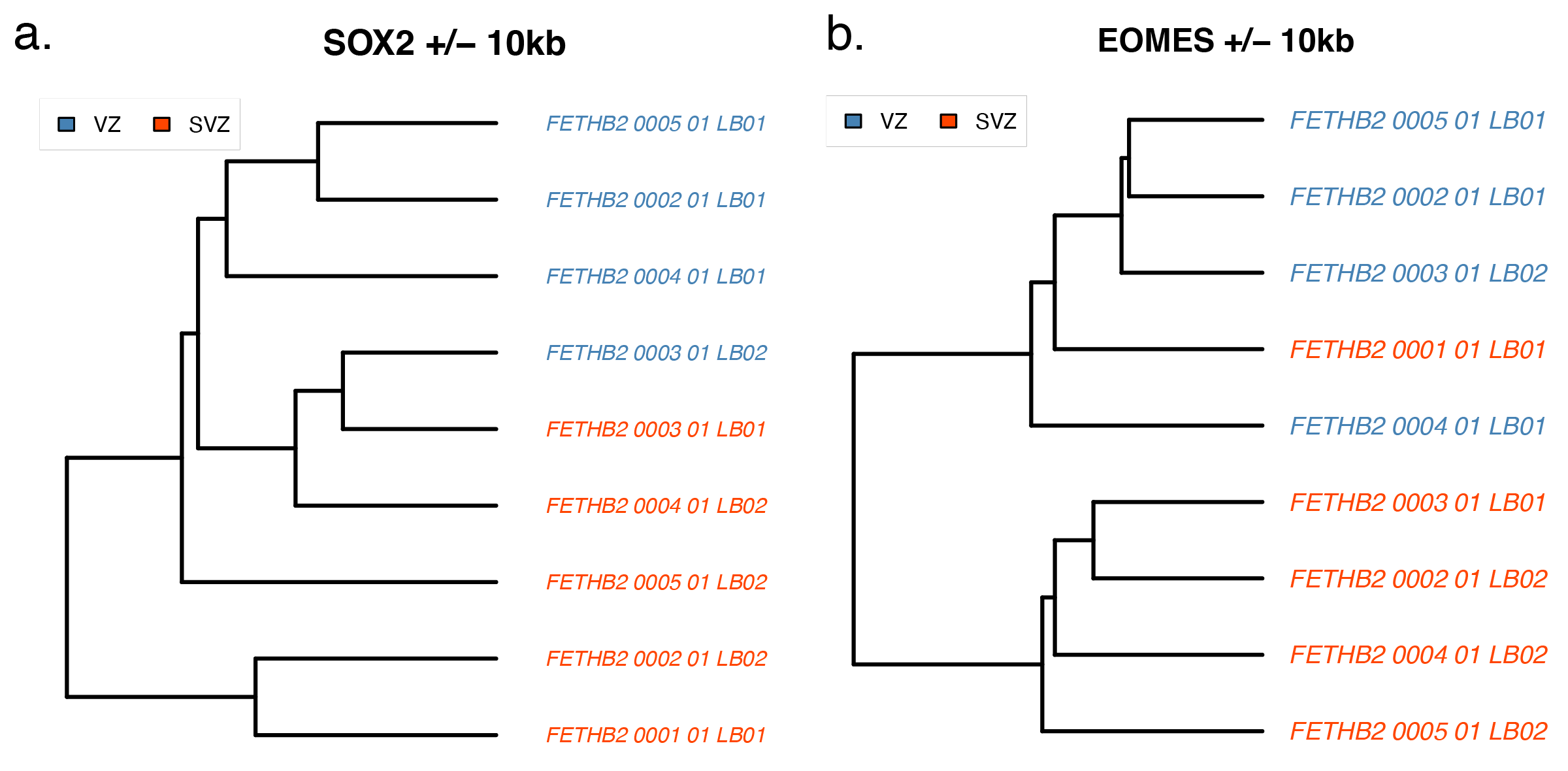
**

**Supplementary Fig. 3.** Hierarchical clustering of DNA methylomes based on CpG methylation of (a) the SOX2 gene region (b) EOMES gene region.

**
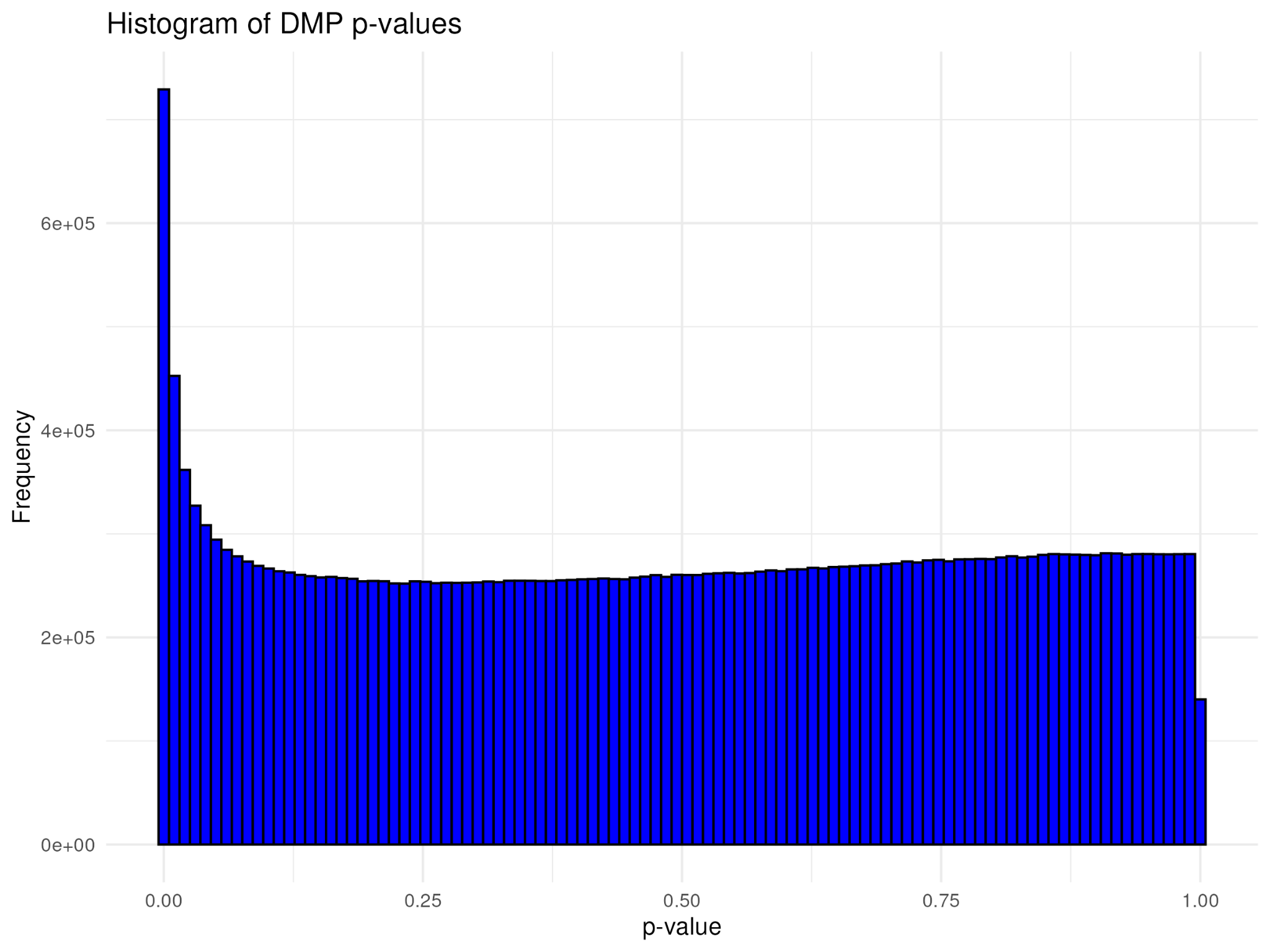
**

**Supplementary Fig. 4.** Distribution of nominal p-values of base-level differentially methylated CpGs between rhombic lip ventricular zone and subventricular zone.


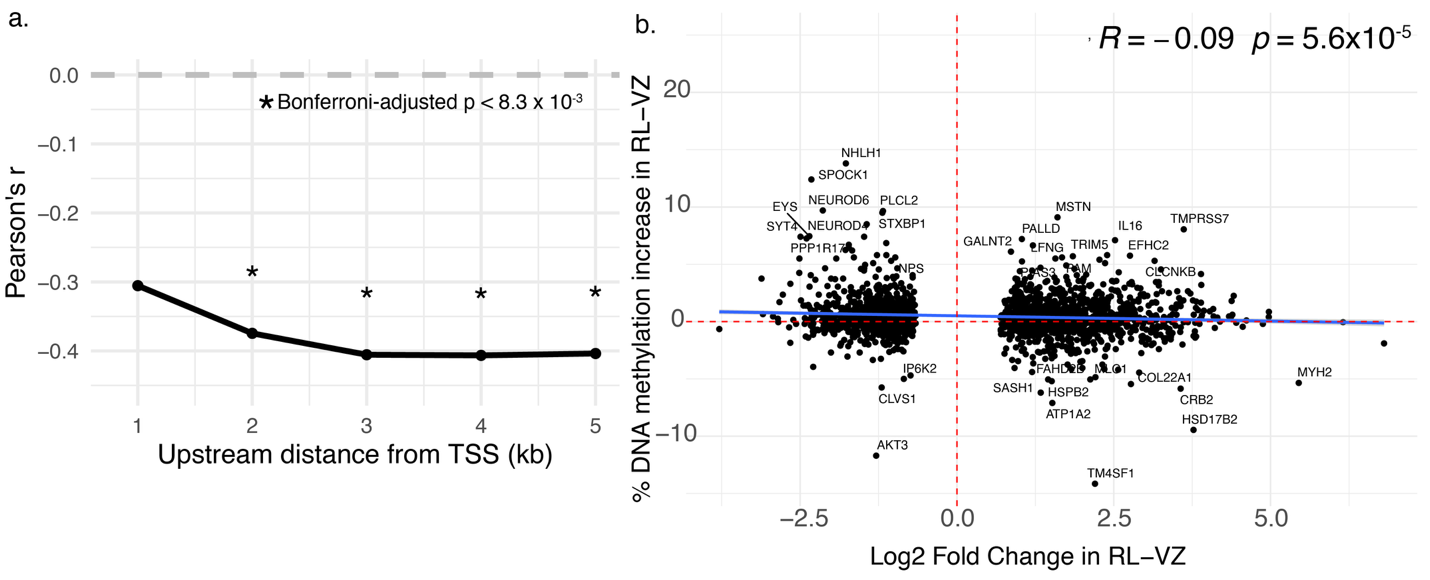


**Supplementary Fig. 5.** Promoter-level DNA methylation and gene expression.

a. Correlation between promoter-level DNA methylation increase in the RL-VZ (DMRs only), and RNA expression change of the corresponding gene in the RL-VZ, for different upstream extents used for the definition of a promoter (x-axis). (N=[58, 61, 64, 65, 68] loci). Correlations that achieved Bonferroni-adjusted statistical significance are indicated with an asterisk.

b. Promoter-level DNA methylation increase and transcription increase of the corresponding gene; analysis limited to genes differentially expressed genes but all genes for which methylation data are available; i.e., not limited to DMRs (N = 2,005 genes). Spearman correlation and corresponding p-value from a t-test are shown.


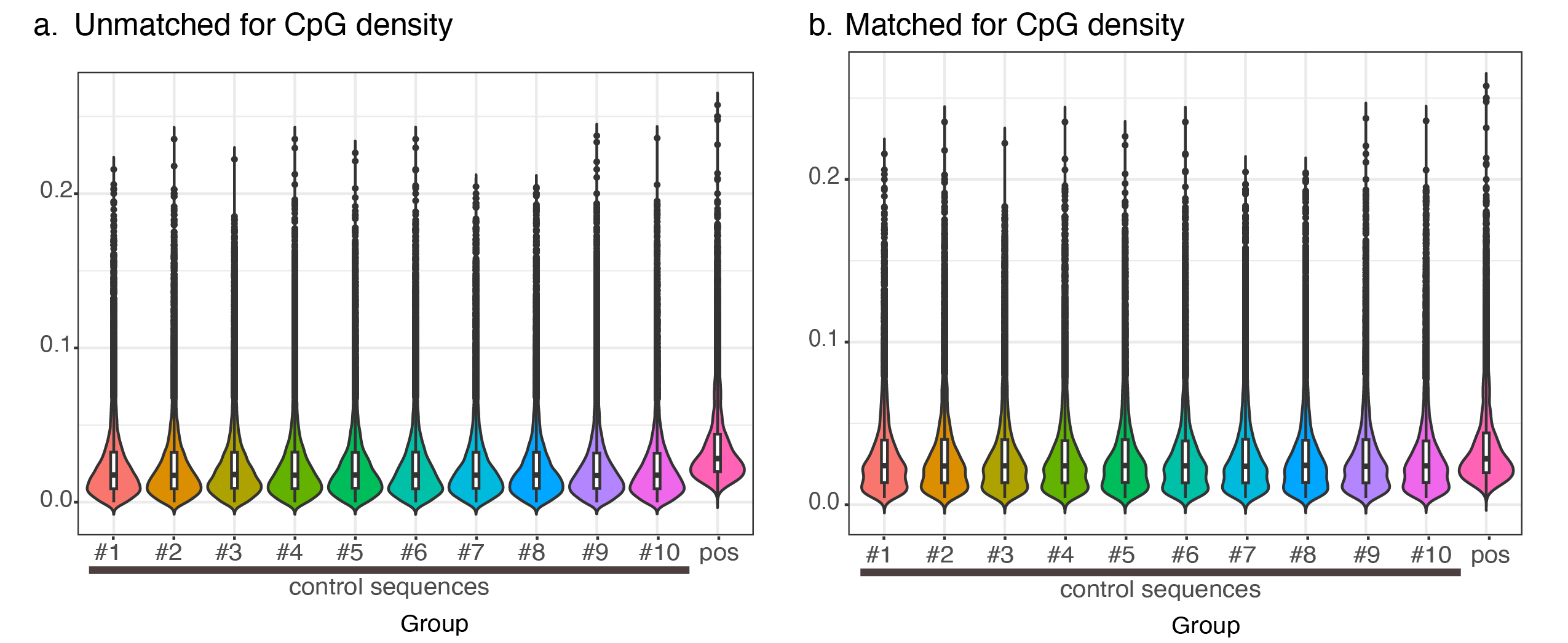


**Supplementary Fig. 6.** Distribution of CpG density in positive sequences and negative control sequences.

a. CpG density in positive sequences and randomly sampled 10 sets of negative sequences, before matching for CpG density. Each violin plot shows data for one set of sequences, with each datapoint showing data for one sequence. N = 9,855 sequences in positive DMR set; overall mean of 8,610 sequences in the 1,000 negative control sets matched for length, GC, and Bismark-mappability.

b. CpG density in the same set of sequences as (a), but after negative sequences were matched to positive sequences for quartiles of CpG density. Overall of 5,862 sequences in the 1,000 negative control sets, after these were additionally subsampled to match for CpG density.

**
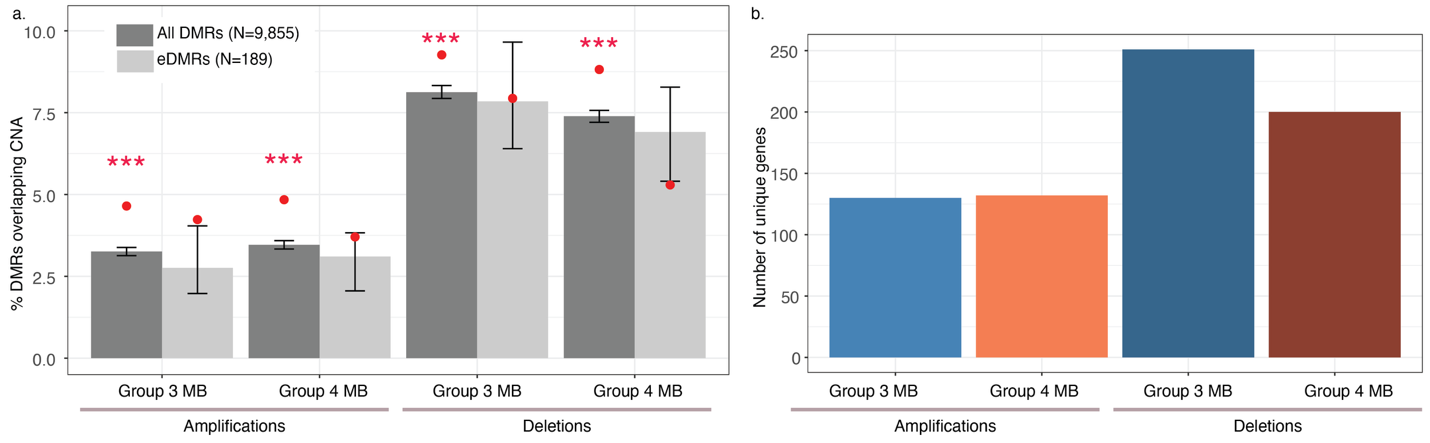
**

**Supplementary Fig. 7.** Overlap of DMRs with copy number aberrations (CNA) in Group 3 and 4 MB genomes.

a. Fraction of real DMRs (red dot) versus control sequences (grey bars) that overlap CNAs. Bar height shows median overlap of 1,000 sets of length-, GC-, and Bismark mappability-matched sequences, while errorbars show interquartile range of the same. Asterisks indicate p < 0.0001.

b. Number of unique genes that overlap DMRs that overlap CNAs.
